# Sir Harold Ridley as the Pioneer of Intraocular Lenses: His Inspiration Drawn From World War II Pilots

**DOI:** 10.7759/cureus.68722

**Published:** 2024-09-05

**Authors:** Kate S Lim, Anuradha Mishra

**Affiliations:** 1 Ophthalmology, Dalhousie University, Halifax, CAN

**Keywords:** general ophthalmology, historical vignette, history of medical sciences, intraocular lens implantation, ophthalmology, ophthalmology education, pioneer in medicine, uncomplicated cataract surgery

## Abstract

This article underscores the monumental contributions of Sir Harold Ridley to the development of intraocular lenses (IOLs), which have revolutionized cataract surgery. Sir Harold Ridley, a British ophthalmologist and medical scientist, drew inspiration from the injuries of World War II pilots to pioneer the first successful IOL implantation in 1949 at St. Thomas’s Hospital. The lens, made from Perspex CQ, marked the inception of modern cataract surgery. Despite facing considerable skepticism and resistance from the medical community throughout the 1950s and 60s, Ridley’s perseverance led to the gradual acceptance of IOLs by the 1970s. Today, Ridley is rightfully recognized as the "father of the intraocular lens," with his groundbreaking work having profoundly impacted the field of ophthalmology and improved the quality of life for millions globally.

## Introduction and background

**Figure 1:**
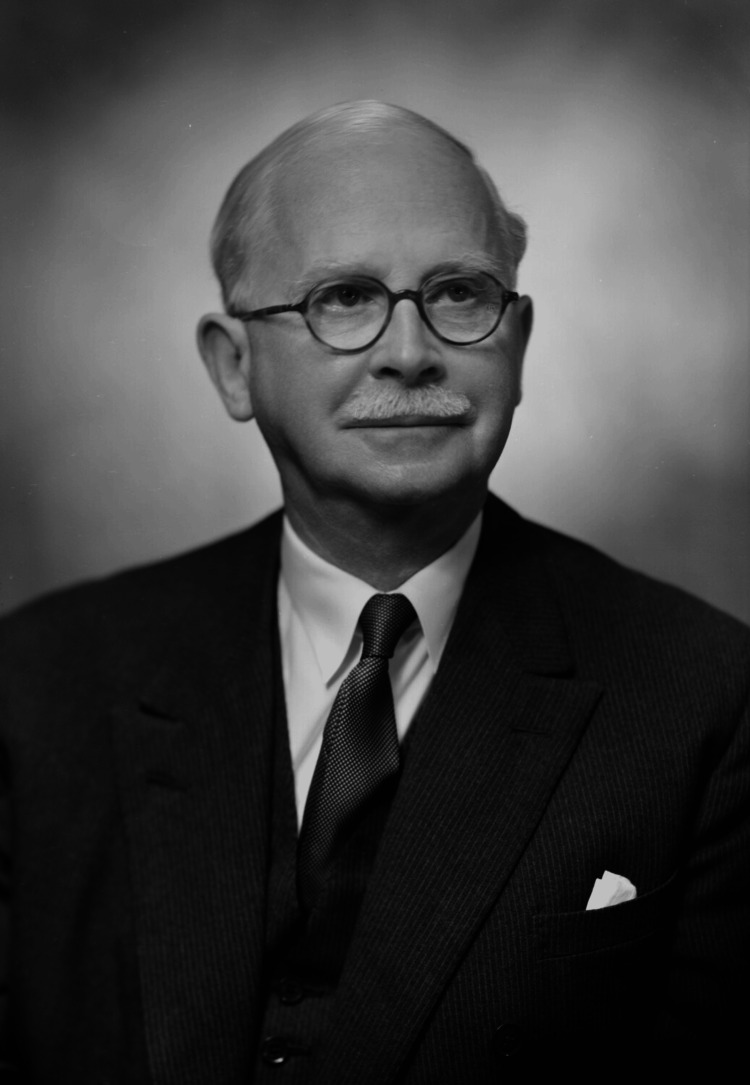
Sir (Nicholas) Harold Lloyd Ridley

The main purpose of this article is to highlight the indisputable contribution of Sir Harold Ridley to intraocular lens (IOL) (Figure 1). Harold Ridley was a British ophthalmology surgeon and medical scientist who utilized his observations and inspirations from the injured pilots of World War II in the creation of the first IOL. In 1949, Ridley performed the first successful implantation of an IOL at St. Thomas’s Hospital [1]. The lens was made from Perspex CQ (clinical quality), a particular monomer cast sheet, and the procedure marked the birth of cataract surgery as we know it today [1]. Ridley's innovation faced considerable skepticism and resistance from the medical community, but he persisted. Throughout the 1950s and 60s, Ridley’s work was not widely accepted. However, his determination paid off as the safety and efficacy of IOLs were gradually recognized. By the 1970s, the procedure began gaining acceptance, revolutionizing the treatment of cataracts. Today, Sir Harold Ridley is often referred to as the "father of the intraocular lens" due to his pioneering work in developing and implanting the first IOL for cataract patients. His innovation has had a profound and lasting impact on the field of ophthalmology, significantly improving the quality of life for millions of people worldwide.

## Review

Early life and education

Harold Ridley was born on July 10, 1906, in Kibworth Harcourt, England [2]. The Ridley family had a strong tradition of service in medicine and the church. Harold's father, Nicholas Ridley, was initially a physician who later pursued a career in ophthalmology after being diagnosed with hemophilia [1], which prevented him from becoming a general surgeon. Despite his health challenges, Nicholas successfully practiced ophthalmology until his death from a cerebral hemorrhage in 1937.

For Ridley, a memorable event in his childhood was meeting Florence Nightingale, the founder of modern nursing and a friend of his mother's, an experience he cherished [3]. Harold's education began at a boarding school in Hove, followed by Charterhouse, a prestigious boarding school near Godalming, Surrey, where he studied from around 1920 to 1924 [3]. He then attended Pembroke College, Cambridge University, excelling in the sciences and earning an honors degree in 1927 [3]. That same year, he commenced his medical training at St. Thomas’s Hospital Medical School in London.

World War II and inspiration

During World War II, Ridley served as an ophthalmologist and was inspired by his work with injured pilots to develop a groundbreaking idea. One significant case involved Flight Lieutenant Gordon 'Mouse' Cleaver [1], who played an inadvertent role in Ridley's innovation. On August 14, 1940, during the pivotal 'Day of the Eagle' battle, Cleaver's cockpit was struck by a bullet [4], shattering the Perspex acrylic canopy. He was blinded in both eyes by multiple fragments of the plastic canopy, leaving him completely blind in one eye and severely impaired in the other [4].

As a result, Ridley performed several surgeries on Cleaver over the years, removing plastic fragments from the eye tissues. Through this work, Ridley observed that the plastic shards were well-tolerated by the eye tissue, causing no inflammation or adverse effects [1]. This observation led him to a revolutionary idea: if the eye could tolerate the acrylic material, it might be possible to create biocompatible artificial lenses from similar materials. These lenses could replace natural lenses in patients with cataracts, significantly improving their vision. This realization paved the way for the development of the IOL, one of the most important advances in eye surgery of the 20th century.

Development of the IOL

After eight years of following up on Flight Lieutenant Cleaver, Ridley's long-held plans to develop an artificial lens, and the corresponding implantation procedure were nearing fruition. With the assistance of a small team, they produced the necessary acrylic material (Perspex, or poly(methyl) methacrylate - PMMA) [1]. Under Ridley's guidance, he worked with a company to synthesize a pure form of this material, called Perspex CQ [1], which remains used for some IOLs today.

In 1949, Ridley performed the first successful implantation of an IOL at St. Thomas’s Hospital [2,5]. The operation, conducted in secrecy, was on a 45-year-old hospital nurse named Elisabeth Atwood, who volunteered for the procedure due to a cataract in one eye [6]. The surgery proceeded without complications and marked a pivotal moment in the history of cataract surgery. This successful procedure demonstrated the feasibility, safety, and effectiveness of IOLs, ultimately revolutionizing cataract treatment and enabling a complete cure for the condition.

Challenges and triumphs

Before 1949, surgeons were primarily trained to remove things from the eye, such as cataracts, blood, pus, and foreign objects. Introducing a foreign material into the eye represented a complete reversal of this practice. This radical shift sparked extensive controversy, with many in the medical community either criticizing or struggling to rationalize the concept [1]. Before Ridley, no one had seriously considered implanting artificial solid or semi-solid substances in the eye.

Unfortunately, Ridley faced significant resistance from the British medical establishment almost immediately after his pioneering work on IOLs became known. Throughout the 1950s and 60s, his work was not widely accepted, and he suffered from severe depression, compounded by the professional jealousy, fear of innovation, and unjustified skepticism he encountered [1]. By the 1970s, the IOL procedure began to gain widespread acceptance, revolutionizing cataract treatment. However, in 1980, the future of the IOL in the United States faced uncertainty when it became the subject of a Food and Drug Administration (FDA) hearing. The FDA may have been concerned that Sir Harold Ridley's original 1949 IOL design and the modifications made over the subsequent decades did not provide enough data for comprehensive analysis [1]. Furthermore, Dr. Sidney Wolfe, the most vocal opponent of the IOL, requested that the US FDA outlaw IOLs, misleadingly citing an overestimation of the IOL complication rate [7]. According to Dr. Wolfe’s statistics, the use of IOLs led to a 50% complication rate; however, this was the result of including even the most minor adverse reactions in the total number of complications [7].

Notably, actor Robert Young, known as "America's Doctor" for his popular television role, had undergone a successful IOL surgery about four years prior. He enjoyed perfect 20/20 vision in both eyes following the procedure and credited the operation with allowing him to continue his acting career. Young stated, “...implants saved my career and should be available to all Americans" [7]. Young's positive experience, along with his testimony at the FDA hearing, helped support the continued use of IOLs. This momentum, combined with the growing body of scientific evidence and support from the 1974 International Congress of Ophthalmology, ultimately solidified the acceptance of IOLs in ophthalmic surgery.

Legacy and honors

Ridley's contributions were eventually recognized, and he received numerous honors later in life. In 1979, he was honored by a group that held great significance for him: the surgeons who had tested and evaluated his invention. This recognition occurred at the 1979 American Academy of Ophthalmology and Otolaryngology meeting in San Francisco, California. During this meeting, he received a book titled, A Salute to Dr. Harold Ridley, which included 4,000+ American ophthalmologists' signatures, recognizing what he had done for their specialty [8]. In 1986, three years after his 80th birthday, Ridley was elected to the Fellowship of the Royal Society [9]. The following year, he was awarded the Gullstrand Medal by the Swedish Society of Medicine [9]. His achievements culminated in 2000 when he was knighted by Queen Elizabeth II for his services to ophthalmology, formally becoming Sir Harold Ridley.

Final years and impact

Sir Harold Ridley passed away on May 25, 2001, at the age of 91, leaving behind a transformative legacy in ophthalmology. His pioneering work not only revolutionized eye care but also made a significant impact on the broader field of medical science. Today, millions of cataract patients benefit from his innovations, and IOL implantation has become one of the most common and successful surgical procedures globally.

## Conclusions

Sir Harold Ridley was a British ophthalmologist who revolutionized cataract surgery by inventing the IOL. After observing injured pilots from World War II who became blind due to fragments of the plastic canopy being lodged in their eyes, he discovered a material that could be safely inserted into the eye. Although he initially faced challenges and resistance in gaining widespread acceptance of IOL implantation within the medical community, his perseverance paved the way for modern cataract surgery. Today, Sir Harold Ridley is recognized as the "father of the intraocular lens," and his invention has had a profound impact on the vision of billions of people globally.
